# Development and Validation of a Combined Methodology for Assessing the Total Quality Control of Herbal Medicinal Products – Application to Oleuropein Preparations

**DOI:** 10.1371/journal.pone.0078277

**Published:** 2013-10-21

**Authors:** Nikolaos Lemonakis, Evagelos Gikas, Maria Halabalaki, Alexios-Leandros Skaltsounis

**Affiliations:** 1 Department of Pharmacognosy and Natural Products Chemistry, Faculty of Pharmacy, National and Kapodistrian University of Athens, Athens, Greece; 2 Department of Pharmaceutical Chemistry, Faculty of Pharmacy, National and Kapodistrian University of Athens, Athens, Greece; National Institutes of Health, United States of America

## Abstract

Oleuropein (OE) is a secoiridoid glycoside, which occurs mostly in the Oleaceae family presenting several pharmacological properties, including antioxidant, cardio-protective, anti-atherogenic effects etc. Based on these findings OE is commercially available, as Herbal Medicinal Product (HMP), claimed for its antioxidant effects. As there are general provisions of the medicine regulating bodies e.g. European Medicines Agency, the quality of the HMP’s must always be demonstrated. Therefore, a novel LC-MS methodology was developed and validated for the simultaneous quantification of OE and its main degradation product, hydroxytyrosol (HT), for the relevant OE claimed HMP’s. The internal standard (IS) methodology was employed and separation of OE, HT and IS was achieved on a C18 Fused Core column with 3.1 min overall run time employing the SIM method for the analytical signal acquisition. The method was validated according to the International Conference on Harmonisation requirements and the results show adequate linearity (r^2^ > 0.99) over a wide concentration range [0.1–15 μg/mL (n=12)] and a LLOQ value of 0.1 μg/mL, for both OE and HT. Furthermore, as it would be beneficial to control the quality taking into account all the substances of the OE claimed HMP’s; a metabolomics-like approach has been developed and applied for the total quality control of the different preparations employing UHPLC-HRMS-multivariate analysis (MVA). Four OE-claimed commercial HMP’s have been randomly selected and MVA similarity-based measurements were performed. The results showed that the examined samples could also be differentiated as evidenced according to their scores plot. Batch to batch reproducibility between the samples of the same brand has also been determined and found to be acceptable. Overall, the developed combined methodology has been found to be an efficient tool for the monitoring of the HMP’s total quality. Only one OE HMP has been found to be consistent to its label claim.

## Introduction

Oleuropein (OE) is a natural secoiridoid glycoside, occuring mainly in the *Olea* genous of the Oleaceae family and it is the most well studied phenolic compound in olive cultivars [1-3]. OE is one of the many individual components of the Mediterranean diet and it has been proved to be exhibiting protective activity against an array of common chronic pathological conditions [4,5]. The molecule has been shown to exert several pharmacological properties, including antioxidant [6], antimicrobial [7], cardioprotective [8,9], anti-ischemic [10], antiatherogenic [11], anti-inflammatory [12,13], antidiabetic [14], antiproliferative in prostate cell lines [15] etc. Thus, several formulations of OE are commercially available in many countries (approximately 23), as food supplements or herbal medicinal products (HMP) [16]. Its main degradation product, which is also a natural occurring substance in olive products, is hydroxytyrosol (HT), which also exhibits several interesting biological properties [17]. 

As HMP is defined “any medicinal product, exclusively containing as active ingredients one or more herbal substances or one or more herbal preparations, or one or more such herbal substances in combination with one or more such herbal preparations” [18]. Furthermore, as stated by the European Medicines Agency (EMA), “irrespective of the regulatory pathway to access the market, the quality of the herbal medicinal product must always be demonstrated” [19]. HMP’s are products of high commercial interest as proved by the wide increase of their use worldwide but due to their high profitability a series of concerns are rising regarding their quality. Thus new reliable and fast quality control methodologies are required for the assessment of HMP’s quality, in order to meet the appropriate standards required for the protection and promotion of public health. It should be noted that the quality control of HMP’s represents a demanding task, as they are usually complex mixtures, extracts or enriched extracts, often containing several hundreds of minor constituents that are impossible to be accurately quantified one by one, taking into account the current status of instrumental capabilities. It is also widely known that synergistic interactions are of vital importance in phytomedicines [20], plant extracts and HMP’s, with one or a few ingredients determining their therapeutic effects but the synergy of all the substances yielding the optimum therapeutic efficacy or possible toxicity on the other hand. In other words, the beneficial effect of a plant extract or HMP cannot be attributed to one single substance, but is the result of the synergistic interplay between many extract's constituents, usually called the multicomponent system. The aforementioned biological activities represent the health claims registered to the corresponding regulatory bodies. Thus it is evident that the quality control of HMP’s as a whole represents an extremely demanding task, as according to the aforementioned concepts, the accurate quantification of each one of several hundreds of minor constituents cannot be performed. On the other hand the measurement of the quality of HMP’s taking into account as many constituents as possible is crucial and at least the batch to batch reproducibility of such products should be demonstrated, as it ensures the same biological activity (due to both the main constituent and the synergistic effects) for the patients receiving the selected commercial formulation. Overall the quality control of the HMP’s as a whole should also be controlled and reported. In the current work the term “total quality” reflects not only the accurate quantitation of the main bioactive substance (OE) but also the simultaneous evaluation of the total phytochemical profile of the investigated OE claimed HMP’s in each formulation. 

Many analytical methods have been developed for the determination of OE in various matrices such as olive oil [21], table olives [22], byproducts [23] and biological fluids [24]. Thus, OE has been analysed employing HPLC-UV [25], HPLC-fluorescence [26] and HPLC-MS/MS [23,27]. Other methods besides liquid chromatography have also been employed such as GC-MS [28], capillary electrophoresis [29] and FT-IR [30]. However no analytical methodologies have been developed and validated for the simultaneous quantification of OE and its main degradation product HT in HMP’s. In order such methodologies applied in industrial environments should exhibit high turnaround time, with minimal sample preparation and fast analysis duration. For this purpose, a novel, rapid and high throughput LC-MS analytical methodology was developed and validated according to the International Conference on Harmonisation (ICH) requirements [31], for the simultaneous quantification of OE and HT in a selected range of OE commercial HMP’s. This methodology allows for the targeted determination of the two analytes in complex mixtures of HMPS’s. As the modern chromatographic trend goes towards fast analyses, the Fused Core column technology [32] has been employed. Such columns can achieve ultra fast separations even under HPLC conditions [33]. In addition, emphasis was given to the isolation of the matrix of the HMP’s, as it was necessary for the correct validation of the analytical methodology. As already referred, HMP’s are highly complex mixtures and thus it is difficult to simulate them artificially, in order to accurately estimate various analytical parameters, such as the matrix effect and the selectivity. For this purpose isolation of the matrix of the HMP’s was performed, using an adsorbent XAD – 7HP resin and the former has been used throughout the validation procedure, e.g. in the same way plasma [34,35] or urine [34] are used in the bioanalytical method validation. The first aim of the current work was to develop and validate a rapid, sensitive and accurate methodology, to quantify simultaneously OE and HT in commercial HMP’s and apply this methodology to selected OE containing HMP’s in order to access their conformity according to their label claim.

Although LC-MS based approaches are widely employed for the targeted quantification of the major bioactive components of the corresponding HMP’s, similar approaches for the evaluation of HMP’s regarding their profiling as well as their total quality control are less common, partly because they are more demanding in terms of instrumentation and data evaluation. It should be noted that hyphenated high resolution techniques such as UHPLC-HRMS (orbitrap or qQToF) are commonly recommended for the profiling of extracts and therefore for the assessment of HMP’s quality control, because they offer high separation ability and simultaneously the ability to record accurate mass data of the all the contained HMP’s substances [36]. Such analytical methodologies in combination with Multivariate analysis (MVA), such as principal component analysis (PCA) or partial least square analysis (PLS), enable the comprehensive assessment of complex HMP’s and sample classification of diverse status, origin or quality in samples. This approach uses the same principles as metabolomics, which is a branch of science that concerns the total metabolome of integrated biological systems as well as the dynamic responses to the alterations of endogenous and/or exogenous factors [37]. A second aim of the present study is to develop a metabolomics-like approach, combining UHPLC-(-ESI)-HRMS and MVA for studying the “total quality” characteristics of the selected HMP’s in terms of their minor component uniformity with the aid to assess their batch to batch reproducibility. 

Overall, in the current study, an HPLC-(-ESI)-MS and an UHPLC-(-ESI)-HRMS approach were developed in order to evaluate the holistic qualities of commercial OE claimed HMP’s, exploring both the quantity of the bioactive compound (OE) in different commercial OE claimed product and the similarity of their phytochemical profiles. 

## Materials and Methods

### Chemicals and Reagents

**Figure 1:**
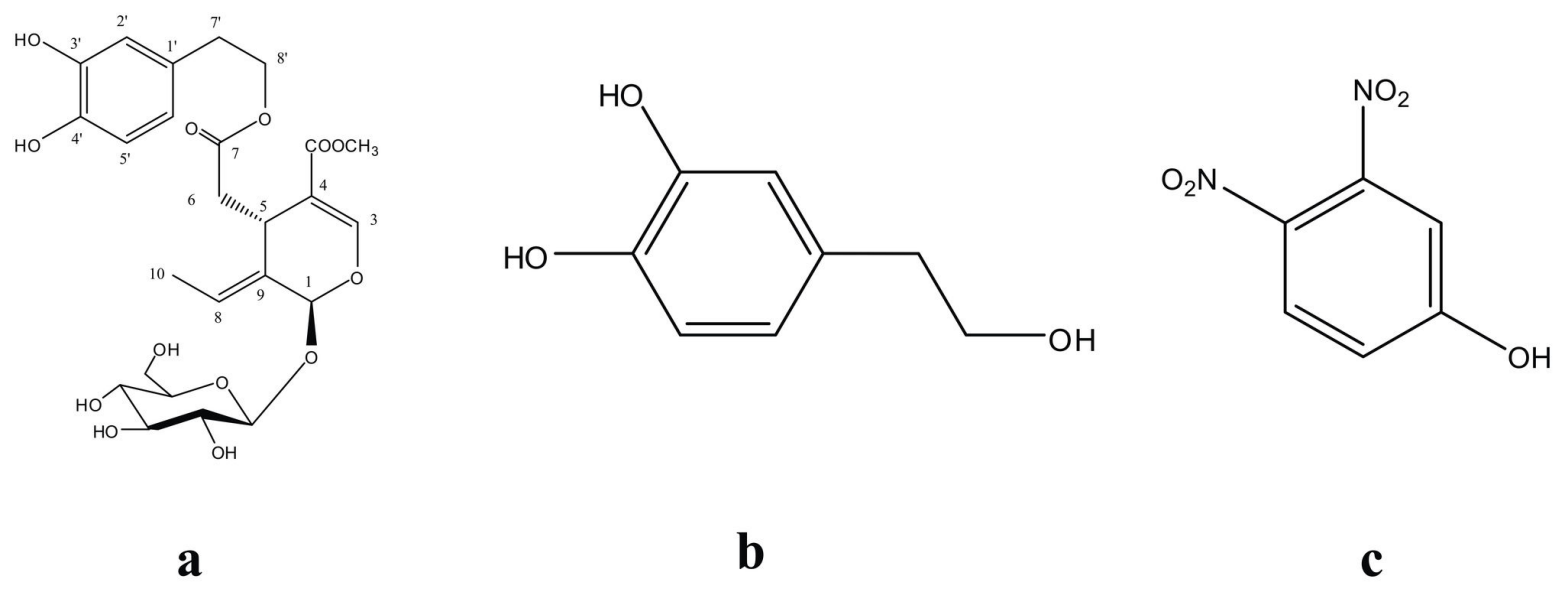
Structures of OE (a), HT (b) and the IS (2,4-dinitrophenol) (c).

The analytical reference standards, OE and HT (Figure 1), were isolated from olive leaves and olive mill wastewater respectively based on previously described procedures [10,38]. No specific permissions were required as the examined plant is an endemic species widely spread in the Mediterranean region, as well as in Australia, the American continent, Africa and Asia. Furthermore *Olea europea* is not included in any list of endangered or protected species. 

Their purity was found to be more than 98% (HPLC-UV) and their structures were identified by High Resolution Mass Spectrometry (HRMS) and 1 & 2D NMR. The internal standard (IS), 2,4-dinitrophenol (Figure 1), was purchased from Sigma – Aldrich (Greece). All solvents used in this study were of LC-MS grade. Acetonitrile (ACN), methanol (MeOH), water and formic acid, were purchased from Fluka/Riedel-de Haën (Switzerland). 2,2-Diphenyl-1-picrylhydrazyl (DPPH) for DPPH experiments was purchased from Sigma – Aldrich (Greece). Absolute ethanol for DPPH dissolving was purchased from Sigma – Aldrich (Greece). The Amberlite XAD-7HP resin used in the absorption experiment was supplied by Sigma – Aldrich (Greece).

### Samples

Five samples (a, b, c, d, e) from eight different production batches (A, B, C, D, E, F, G, H) over a year of Brand1 (B1) were analyzed. B1A batch, which was a special preparation for further biological experiments, was containing a higher amount of OE. All the other samples have been provided by the local market. Thus five samples (a, b, c, d, e) from each of three different vendors (two European and one Chinese) of OE claimed HMP’s have been acquired from the local market. [Brand2 (B2), Brand3 (B3), Brand4 (B4)]. A list of the samples used for the analysis is tabulated in Table 1.

**Table 1:** A summary of tested samples.

Brands (B)	Brand1 (B1)	Brand2 (B2)	Brand3 (B3)	Brand4 (B4)
Batches (n=8)	B1Aa…e*	B2Aa…e	B3Aa…e	B4Aa…e
	B1Ba…e	-	-	-
	B1Ca…e	-	-	-
	B1Da…e	-	-	-
	B1Ea…e	-	-	-
	B1Fa…e	-	-	-
	B1Ga…e	-	-	-
	B1Ha…e	-	-	-

* The following coding has been used. BxYz, x= brand, Y= batch, z= sample number

### Equipment

Nuclear Magnetic Resonance (NMR) spectra were recorded with the aid of a Bruker Avance III spectrometer operating at 600.11 MHz (Bruker Biospin GmbH, Reinsteten, Germany). HPLC analyses for the quantification study were performed employing a Surveyor system (Thermo Scientific, Germany) equipped with a binary pump, an autosampler, an online vacuum degasser and a temperature-controlled column compartment. Mass spectrometry for the OE and HT quantitation was performed on a single quadrupole MSQ mass spectrometer (Thermo Scientific, Germany) connected to the HPLC system. UHPLC for the quality control - multivariate analysis study was performed employing an Accela system (Thermo Scientific, Germany) equipped with a binary pump, an autosampler, an online vacuum degasser and a temperature-controlled column compartment. High-resolution mass spectrometry was performed on a hybrid LTQ Orbitrap Discovery XL mass spectrometer (Thermo Scientific, Germany). Centrifuging was performed by a Mikro 200R centrifuge (Hettich Lab Technology, Germany). Evaporation of the samples was performed with the aid of a Buchi evaporator system consisting of a rotavapor R-210, a vacuum pump V-700 and a vacuum controller V-850 (Buchi, Switzerland). A micro plate Reader, Infinite M200^®^ PRO (Tecan, Austria) was used for the evaluation of the DPPH activity of the capsules.

### Simultaneous quantification of OE and HT in HMP’s – Validation of the analytical methodology

#### LC-MS Conditions

 An Ascentis Express RP-C18 Fused Core (100 x 2.1 mm, 2.7 μm) column (Supelco Analytical, Germany) was used at a flow rate of 0.3 mL/min for the chromatographic separation. The mobile phase consisted of: solvents A - water, 0.1% formic acid and B - ACN. A gradient methodology (total run time of 3.1 min) was used for the separation of OE, HT and the IS as follows: 0 to 0.5 min: 90% A: 10% B, 0.5 to 0.6 min: 70% A: 30% B, 0.6 to 1.5 min: 70% A: 30% B, 1.5 to 2.5 min: 30% A: 70% B, 2.5 to 3.1 min: 90% A: 10% B. The column temperature was kept at 30 °C throughout all experiments and the injection volume was 10 μL.

The mass spectrometer parameters (source temperature, dwell time, cone voltage) were optimized for the selected ion monitoring (SIM) mode in order to achieve optimal sensitivity and selectivity (Table 2). Thus the mass spectrometer (MSQ) was operated in the negative ion mode under the following conditions: ESI voltage, -4500 V; source temperature, 450°C. All compounds were analyzed as their respective deprotonated ions [M-H]^-^. 

**Table 2:** Mass spectrometry parameters - SIM conditions.

MS	Name	Mass	Time (min)	Dwell Time	Cone (V)
SIM	HT	153	0-3.1	0.07	65
SIM	IS	183	0-3.1	0.07	65
SIM	OE	539	0-3.1	0.07	75

#### Preparation of the matrix

Initially, 2.1 g of capsules content were dissolved in 100 mL of HPLC grade water and sonicated for 5 min. The preparation of the resin was as follows: 150 g of XAD – 7HP resin were placed in a separation funnel, washed by 100 mL HPLC grade water, in order to remove any suspended solids and subsequently the resin was regenerated by concurrent feeding of 100 mL MeOH. In a final step, the resin was washed with 150 mL HPLC grade water prior to its use. The aforementioned capsule’s content solution (100 mL) was loaded onto the XAD – 7HP resin. The eluate was collected and the solvent was evaporated using a rotary evaporator, affording a dry residue of 1.5 g. The absence of OE and HT was confirmed by LC-MS analysis employing the developed quantitation methodology. Thus, this eluate has been used as matrix throughout the validation procedure. 

#### Preparation of standard solutions, calibration curve and quality control (QC) samples

14.57 mg of matrix was suspended in 2.43 mL MeOH, followed by brief vortexing affording a suspension of 6 mg/mL. Consequently the suspension was sonicated for 5 min, centrifuged (12,000 rpm) for 3 min, and the supernatant was transferred into a glass tube and diluted 1/100 (v/v %) in aq. 0.1% formic acid/ACN 90/10 (v/v %) giving a solution 60 μg/mL of the matrix (Matrix-Matched Solution, MMS). An aliquot was transferred to an appropriate vial (1.8 mL) for LC-MS analysis. The MMS was used throughout all the validation procedure. Stock solutions of analytes, OE, HT and IS, were prepared at concentration of 1 mg/mL in MeOH and stored in the dark at 4 °C. Working solutions were daily prepared by diluting appropriate volumes of stock solutions in MMS to obtain 100 μg/mL solutions for each analyte of interest. The IS working solution was prepared by diluting an appropriate volume of the corresponding stock solution in MMS, in order to obtain a 20 μg/mL concentration level. The calibration curves covered the range 0.1 - 15 μg/mL for both analytes, whereas the concentration of IS was 2 μg/mL. Different sets of solutions were prepared specifically, in order to be used as QC samples. QC samples were prepared in the same way at three concentration levels, a low (LQC 4.0 μg/mL), a medium (MQC 9.0 μg/mL) and a high (HQC 12.5 μg/mL). 

#### Sample Preparation – Extraction Protocol

A total of fifty-five capsules, 5 from each brand and/or batch were processed (see Table 1). Samples were prepared for LC-MS analysis according to the following protocol: The content of each capsule was suspended in MeOH followed by brief vortexing, affording a suspension of 6 mg/mL. The samples were sonicated for 5 min, subsequently centrifuged (12000 rpm) for 3 min, and the supernatant was transferred into glass tubes and diluted 1/100 (v/v) in aq. 0.1% formic acid/ACN 90/10 (v/v). An aliquot of the samples at the expected concentration level of 60 μg/mL was transferred to appropriate vials for analysis by LC-MS.

#### Quantification

Quantification was performed employing the SIM negative ion mode for OE (MW: 540 g/mol), HT (MW: 154 g/mol) and IS (MW: 184 g/mol). The ion beams (*m/z* 539 for OE, *m/z* 153 for HT and *m/z* 183 for IS) were optimized by adjustment of the cone voltage, the probe temperature and dwell time for each compound. An FWHM value of 0.7 was used throughout all quantification experiments. The IS methodology has been employed for the quantification of both analytes based in the calculation of the peak – area ratios of each analyte to that of IS. 

#### Validation of the analytical methodology

The method validation was performed in accordance to the International Conference on Harmonisation (ICH) Q2 (R1) guidelines by evaluating stability, specificity, linearity, precision, accuracy, quantitation and detection limit, robustness and system suitability parameters [30].

#### Specificity – Recovery - Matrix Effect

Specificity has been examined by the injection of six individually prepared MMS solutions and the presence of possible interferences at the corresponding t_R_ of the analytes and the IS was investigated. The matrix effect was evaluated comparing the slope of two calibration curves, one prepared in aq. 0.1% formic acid/ACN 90/10 (v/v) and the other prepared in MMS, as described above. Recovery was determined at the three different QC levels (LQC, MQC and HQC), by comparing the concentration of analytes determined for matrix samples (n=5) spiked with the analytes before the extraction (samples prepared by spiking the MMS in the dry state with the appropriate volumes from the standard stock solutions of analytes and the IS and air dried in room temperature in order to remove the solvent), with the corresponding areas of MMS samples spiked with the target analytes at the same concentration post-extraction. The same procedure was employed for the determination of the IS recovery. 

#### Regression model

Calibration curves ranging from 0.1 to 15 μg/mL for both analytes of interest were constructed and analyzed on three separate days and fitted using linear regression analysis employing 1/x weighting. The 0.0 point was neither included as a point nor the calibration curve was forced to pass through it. 

#### Quantitation and detection limit

The LOQ was calculated comparing measured analyte signals with those of blank samples (samples not spiked with analytes) and establishing the minimum concentration at which the analytes can be reliably quantified leading to a typical signal-to-noise ratio of 10:1. The LOD was calculated in the same manner by comparing measured analyte signals from samples with known low concentrations to those of blank samples and establishing the minimum concentration at which the analytes can be reliably detected with a signal-to-noise ratio of 3:1. 

#### Repeatability, intermediate precision and accuracy

Five replicates of each QC sample at the three QC concentration levels (LQC, MQC, HQC) as well as at the limit of quantitation (LOQ) and upper limit of quantitation (ULOQ), were determine in order to evaluate the repeatability and the intermediate precision of the assay. The results were expressed as the standard deviation (SD) and the relative standard deviation (%RSD) for all QC, LOQ and ULOQ samples. Accuracy was determined for the same samples as the % standard error (% Er) between the mean concentrations calculated and the nominal concentrations. 

#### Robustness

For the establishment of the method’s robustness, experimental conditions were deliberately altered and several parameters were calculated. This was achieved by altering three conditions by ±5%: setting the flow rate of the mobile phase at 0.285 mL/min and 0.315 mL/min, the column temperature at 28.5 °C and 31.5 °C and the capillary temperature at 427.5 °C and 472.5 °C. All experiments were performed at the MQC level (n=5) calculating the chromatographic peak area of each analyte, the retention time (t_R_), the 10% peak asymmetry, the 10% peak width and the USP theoretical plates number. The results were expressed as %RSD.

### Total quality control of HMP’s

 The same samples (section 2.2) have also been used for the total quality control of HMP’s.

#### UHPLC-HRMS Analysis

A Hypersil Gold C18 50 x 2.1, 1.9 μm reversed phase column (Thermo Scientific, Germany) was used at a flow rate of 0.35 mL/min for the chromatographic separation. The mobile phase consisted of solvents A: water, 0.1% formic acid and B: ACN. A gradient method (total run time of 11 min) was used for the profiling of the samples as follows: 0 to 8 min: 95% A: 5% B, 8 to 8.5 min: 60% A: 40% B, 8.5 to 9 min: 5% A: 95% B, 9 to 9.5 min: 95% A: 5% B, 9.5 to 11 min: 95% A: 5% B. Column temperature was kept at 30 °C throughout all experiments and the injection volume was 4 μL.

A hybrid high resolution mass spectrometer (LTQ-Orbitrap Discovery) operating in negative ion mode under the following conditions: capillary temperature, 430°C; capillary voltage, -60 V; tube lens, -90 V; source voltage, 3.30 kV; sheath gas flow, 50 arb. units; aux gas flow, 30 arb. Units has been employed. Analysis was performed in the full scan ion mode, using a resolution of 30000. 

#### DPPH radical scavenging assay

The antioxidant capacity of the OE claimed HMP’s was measured employing the DPPH radical scavenging method [39]. Briefly, a DPPH solution was prepared daily dissolving 12.5 mg in 100 mL of absolute ethanol and stored at 4 °C in the dark until use. All tested HMP samples were diluted in MeOH up to a concentration level of 6 mg/mL. A 7 μL aliquot of each HMP sample solution was added to 193 μL of the DPPH solution into a 96-wellplate to complete the final reaction. After 30 min at room temperature in the dark, the absorbance was measured at 517 nm in a micro plate spectrophotometer reader. The DPPH–ethanol solution with the addition of 7 μL MeOH was used as a control sample. Gallic acid was used as a reference for the DPPH radical scavenging method. Measurements were performed in triplicate using the Magellan software (Tecan, Austria) and the results were expressed as mean ± %RSD. 

#### Data Analysis

Data pre-processing for the multivariate statistical analysis was performed using the MZmine – 2.2 software (http://mzmine.sourceforge.net). The procedures of peak detection, deconvolution, normalization, deisotoping and alignment have been applied to data directly imported as raw files from the mass spectrometer. The generated peak list (accurate mass - retention time *vs* intensity) has been exported and manipulated to Excel using the CONCATANATE, ROUND and TRANSPOSE commands. The generated .xls file has been exported to SIMCA P+ 10.5 (Umetrics, Umea, Sweden) for the multivariate analysis.

#### Principal Component Analysis

PCA has been performed using both unit variance and Pareto scaling; confidence level on parameters was set at 95% whereas 200 maximum iterations have been employed. The scores values have been used for the evaluation of the results. The R^2^ value has been used in order to evaluate the optimal number of contributing parameters. A cut-off value of 10% explained variance has been used for the optimal number of pc’s kept for the construction of the PCA model. 

## Results and Discussion

**Figure 2:**
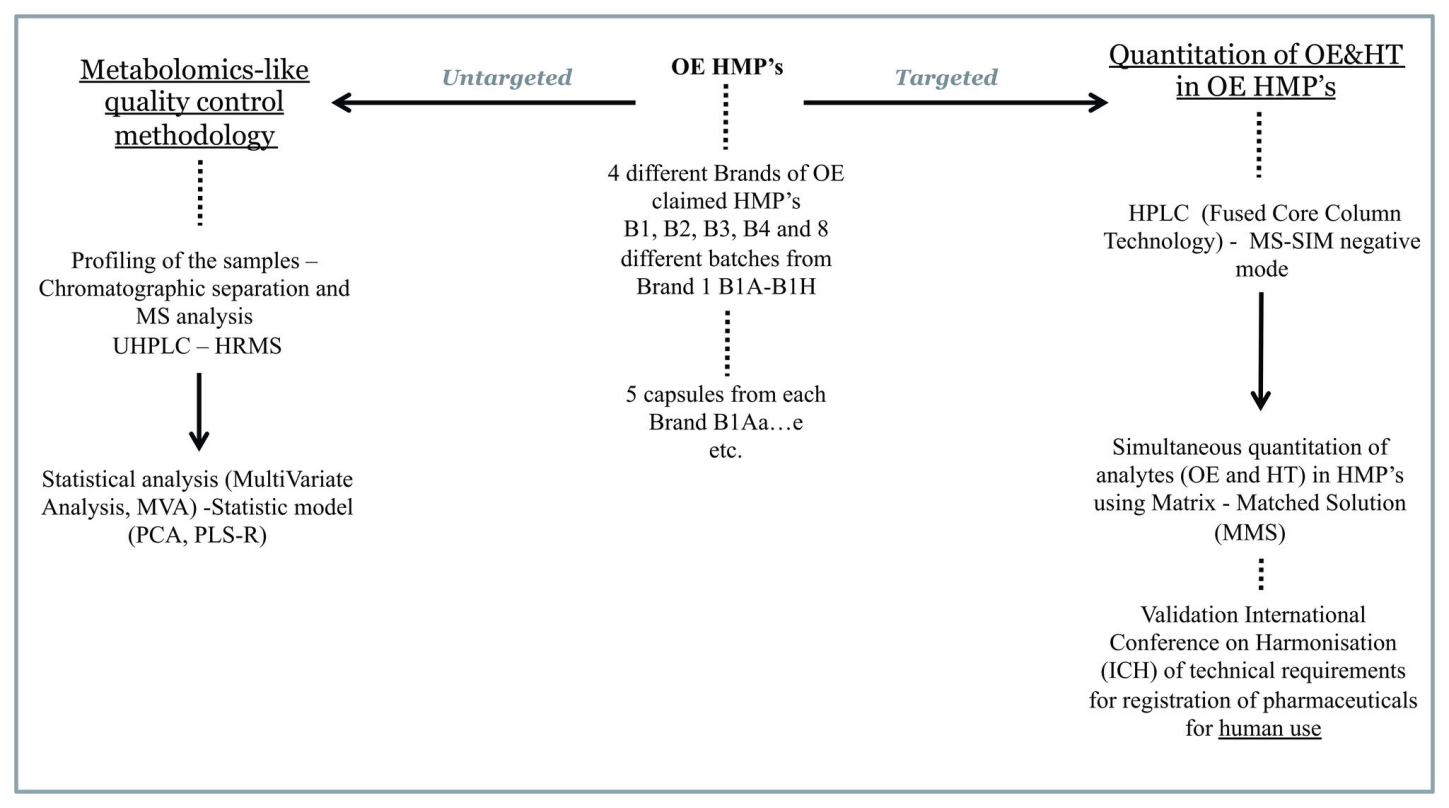
Workflow of the developed total quality control methodology. The right part of the figure corresponds to quantification of OE and HT in selected OE claimed HMP’s and the left part corresponds to the metabolomics-like quality control methodology of the selected OE claimed HMP’s.

An outline of the described methodology is depicted in Figure 2. 

### Simultaneous quantification of OE and HT in HMP’s – Validation of the analytical methodology

#### LC-MS conditions

Chromatographic conditions were analyzed by varying parameters such as mobile phase, gradient elution type, flow rate, type of column chemistry (C18 & C18-NPS, C8, and Phenyl) and column geometries (250 x 4.6 mm, 5μm; 100 x 2.1 mm, 5μm; 50 x 2.1 mm, 5μm, 100 x 2.1 mm, 2.7 μm). Employing the described methodology (section 2.4.1) on an Ascentis Express C18 (Fused Core) 100 x 2.1 mm, 2.7 μm reversed phase column, sharp peaks were achieved leading to enhanced signal intensity and adequate resolution of the analytes from possible interfering components. Furthermore the total analysis time did not exceeded 3.1 min affording a rapid chromatographic methodology. Resolution between all the chromatographic peaks was greater than 2, which ascertains that all analytes are baseline separated. 

Mass spectroscopic parameters (Table 2) were optimized in order to achieve the highest signal for all compounds of interest. In addition, optimization of scan and dwell time afforded well described peaks and highly stable signals. 

#### Matrix Preparation

**Figure 3:**
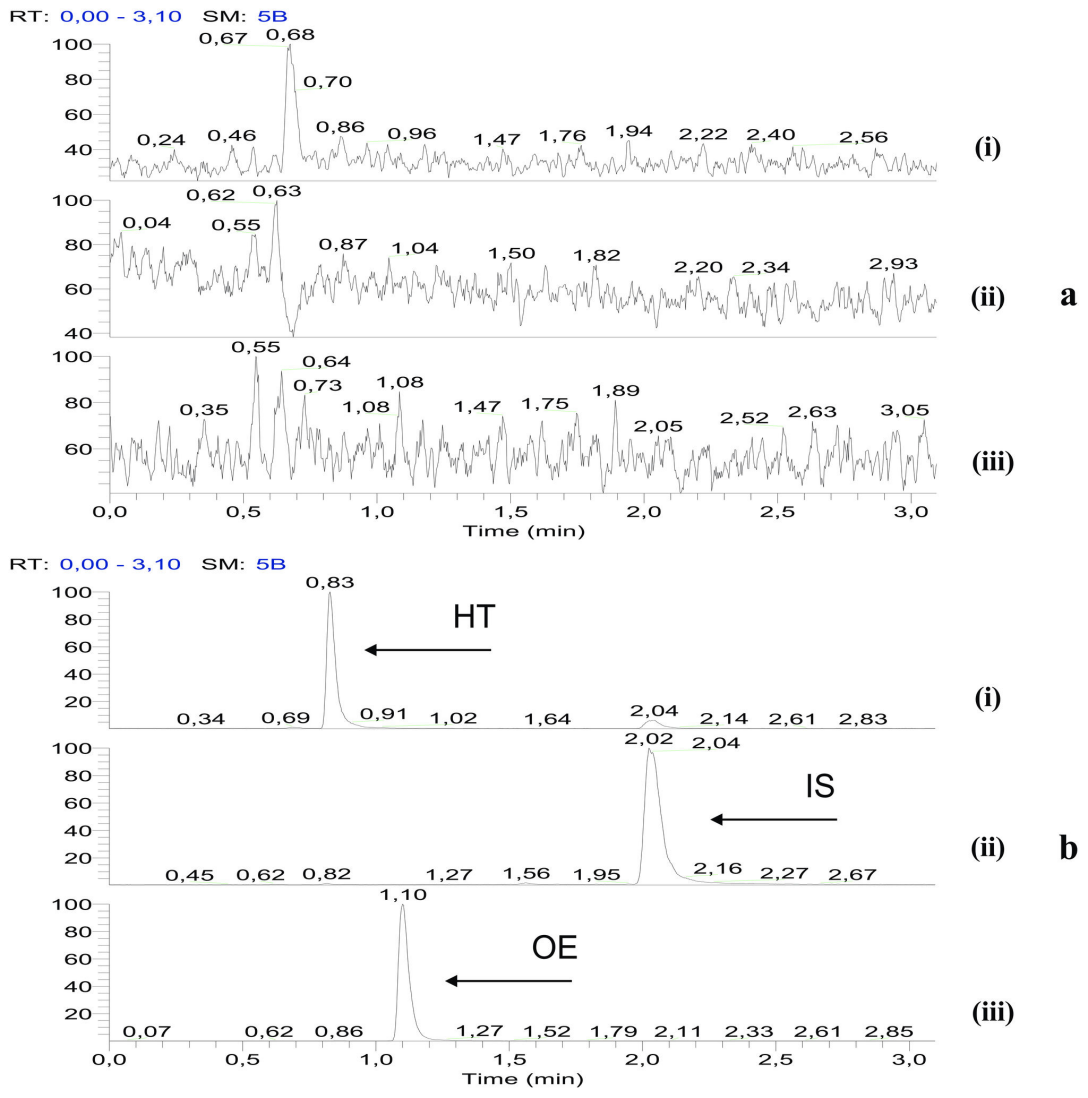
Typical chromatograms of the targeted quantitation of OE and HT using the proposed methodology. a. LC-SIM chromatogram of matrix used as the blank solution (MMS), b. LC-SIM chromatogram of the MMS spiked with OE, HT and IS at 2 μg/mL. The ions monitored were m/z 152.5-153.5 for HT (i), m/z 182.5-183.5 for the IS (ii) and m/z 538.5-539.5 for OE (iii) respectively.

Isolation of the matrix was achieved following the described methodology (section 2.4.2) and the absence of OE and HT was confirmed using the developed LC-MS method. A typical LC-SIM chromatogram of matrix used as the blank solution is shown in Figure 3a. The adsorbent XAD – 7HP resin can selectively retain the polyphenolic content of the extract (capsules) whereas the remaining compounds (matrix) are eluted during the procedure. As the OE and HT represent the main polyphenolic compounds of this extract (olive leaf extract), it is anticipated that the resulting eluate i.e. the matrix used, closely resembles the initial extract (capsule). 

#### Sample Preparation – Extraction Protocol

For the efficient cleanup of the capsule samples and the isolation of the analytes, many different protocols were evaluated for the sample preparation, such as solid phase extraction and solid - liquid extraction with various solvents such as MeOH, ethanol, propanol and water. Solid - liquid extraction with MeOH was chosen as it ensured good extraction efficiency and high reproducibility for all analytes (OE and HT) and the IS and no interference from many co-eluted components of the capsules, whereas the analysis cost and the corresponding time remained at a minimum in terms of sample preparation. 

#### Quantification

Quantification was performed in the SIM negative ion mode for OE, HT and IS. In this study, 2,4-dinitrophenol was chosen as the IS for the LC-MS method since it exhibited high and repeatable recovery and chromatographic behavior with the analytes, it has been shown to be stable under the described experimental conditions (chemical and signal stability), and it did not interfere with the analysis of the compounds of interest. Figure 3b shows chromatograms for the MMS spiked with OE, HT and IS at the 2 μg/mL level. Blank extracts (MMS) that were processed using the described methodology, produced no discernible peaks for OE, HT and IS (shown in Figure 3b). 

#### Specificity – Recovery - Matrix Effect

The specificity was evaluated and no interference was found to the corresponding t_R_ of the analytes and the IS. The matrix effect was evaluated by comparing the slopes of two calibration curves, one prepared in aq. 0.1% formic acid/ACN 90/10 (v/v) and the other prepared in MMS, as described above. The slope ratios were found to be 0.85 and 0.94 for OE and HT respectively. The data exhibit that the analyte signal does not essentially alter when the MMS was used.

Recovery was determined at the three different QC levels (LQC, MQC and HQC) and calculated as %R=pre spiked concentration of analyte/post spiked concentration of analyte x 100. High recovery rates render the method suitable for the analysis of OE and HT in the selected preparations (B1, B2, B3 and B4).

#### Regression model

Calibration curves of OE and HT were linear over the range 0.1 - 15.0 μg/mL, with a correlation coefficient (r^2^)>0.99. Typical equations for the calibration curves are shown in Table 3 and were used for the quantitation of OE and HT in OE claimed HMP’s. Performing a t-test it has been also shown that the intercept was not significantly different from 0, at the 95% confidence level.

**Table 3:** Typical equations for the calibration curves of OE and HT.

Analytes	Equations	R^2^	Weighting
OE	C_OE_ = 2.3 10^-1^ (± 4.3 10^-4^) Area Ratio – 5.3 10^-2^ (± 3.0 10^-2^) R^2^ = 0.997 W: 1/X	0.997	1/X
HT	C_HT_ = 3.1 10^-2^ (± 4.5 10^-4^) Area Ratio – 3.0 10^-4^ (± 3.1 10^-4^) R^2^ = 0.9993 W: 1/X	0.9993	1/X

#### Quantitation and detection limit

The LOQ of both for OE and HT was found to be 0.1 μg/mL and is determined with acceptable accuracy and precision at this concentration level according to the ICH regulations. The LOQ for both analytes defined as the concentration that yielded a peak with a signal/noise ratio of 10 and at least 3 times the response compared to that blank extracts (samples not spiked with analytes). Furthermore the accuracy, expressed as the relative percentage error %Er was found to be lower than 3.8% whereas the precision expressed as the %relative standard deviation %RSD was found to be less than 14.9% for both analytes at the LOQ level. The data are shown in Table 4. The LOD was found to be 0.03 μg/mL for both analytes. The data shows that the described method is of acceptable sensitivity for the analysis of OE and HT in formulations.

**Table 4:** Validation results of the proposed methodology.

(n=5)	Repeatability		Intermediate Precision		Accuracy	
Nominal Value (μg/mL)	OE [Mean ± SD^a^, (%RSD^b^)]	HT [Mean ± SD^a^, (%RSD^b^)]	OE [Mean ± SD^a^, (%RSD^b^)]	HT [Mean ± SD^a^, (%RSD^b^)]	OE % Std Error^c^	HT % Std Error^c^
ULOQ	14.6 ± 0.7 (4.0)	14.9 ± 0.2 (1.0)	14.7 ± 0.6 (4.1)	16.1 ± 1.4 (9.3)	15.0	10.1
HQC	13.0 ± 0.8 (5.2)	12.0 ± 0.3 (2.3)	12.0 ± 0.4 (3.7)	12.6 ± 0.7 (5.2)	12.5	1.7
MQC	9.1 ± 1.4 (14.9)	9.0 ± 0.3 (3.1)	9.2 ± 0.4 (4.0)	8.9 ± 0.5 (5.3)	9.0	-1.3
LQC	4.1 ± 0.1 (3.7)	3.9 ± 0.1 (2.3)	4.3 ± 0.2 (3.8)	4.2 ± 0.3 (7.9)	4.0	0.3
LOQ	0.10 ± 0.01 (8.4)	0.11 ± 0.02 (14.4)	0.1 ± 0.01 (14.9)	0.11 ± 0.02 (18.9)	0.1	3.8

^a^Standard deviation, ^b^% Relative standard deviation, ^c^% Standard error.

#### Repeatability, intermediate precision and accuracy

Repeatability, intermediate precision and accuracy were determined by analyzing replicates (n=5) at the three QC concentration levels (LQC, MQC and HQC), as well as at the LOQ and ULOQ level. The repeatability derived from all QC levels as well as LOQ and ULOQ, did not exhibit values more than 14.4% for OE and more than 14.9% for HT respectively. The intermediate precision did not exhibit values more than 14.9% for OE and 18.9% for HT respectively for all QC, LOQ and ULOQ levels. The %Er derived was found to be within ±10.1% for OE and ±2.7% for HT. The results of accuracy and precision of the method for the two analytes are summarized in Table 4 and proved that the described method can reliably use for the quantitation of OE and HT in OE claimed HMP’s.

#### Robustness

To ensure the insensitivity of the HPLC method to minor changes in the experimental conditions, it is important to demonstrate the robustness of the analytical method. None of the alterations caused a significant change more than 2% for all the parameters examined, proving that the method is sufficiently robust to be used for quality control measurements. Thus, %RSD of the resolution between OE and HT was found to be less than 2% when the conditions were deliberately altered according to the robustness scheme. Changing the flow rate of the mobile phase from 0.300 mL/min to 0.285 mL/min and 0.315 mL/min the %RSD of the resolution between peaks did not exceed 3.4%. It was also noticed that changing the column temperature from 30 °C to 28.5 °C and 31.5 °C, the %RSD of the resolution between peaks was found to be less than 1.5%. Furthermore, by altering the capillary temperature from 450 °C to 427.5 °C and 472.5 °C the peak area for all peaks was not significant affected.

#### Measurement of OE and HT in HMP’s

Quantification of OE and HT in HMP’s was based on standard curves that were prepared daily. From the analysis of the results it has been found that only one brand, i.e. B1, was precise concerning the content of OE in accordance to its label claim. In one brand, B4, neither OE nor HT were found, whereas two brands, B2 and B3, were found to be out of their label claim. The content of OE and HT (mg/Cap) in the examined OE claimed capsules from different brands are shown in Table 5 and in Table S1.

**Table 5:** Assayed OE and HT capsule content from the different HMP’s analyzed.

n=5	OE		HT	
	Mean (mg/cap)	%RSD^a^	Mean (mg/cap)	%RSD^a^
Brand 1 (300 mg cap)	51,1	2,5	1,8	0,6
Brand 2 (150 mg cap)	8,9	0,6	NF	-
Brand 3 (450 mg cap)	26,1	1,0	NT	-
Brand 4 (200 mg cap)	NF	-	NF	-

^a^ % Relative standard deviation.

NF Not Found

NT Not Trusted

### Total quality control of HMP’s

#### Validation of UHPLC-HRMS analysis

In order to assess the reproducibility of the method, ten QC identical samples were analysed at the beginning and at the end of the procedure. The variation of t_R_ was less than 0.5% RSD and the variation of accurate mass was less than 3 ppm, demonstrating the excellent reproducibility of the procedure. Peak area RSD was found to be lower than 6.2% for all the analytes. 

#### Antioxidant activity

**Figure 4:**
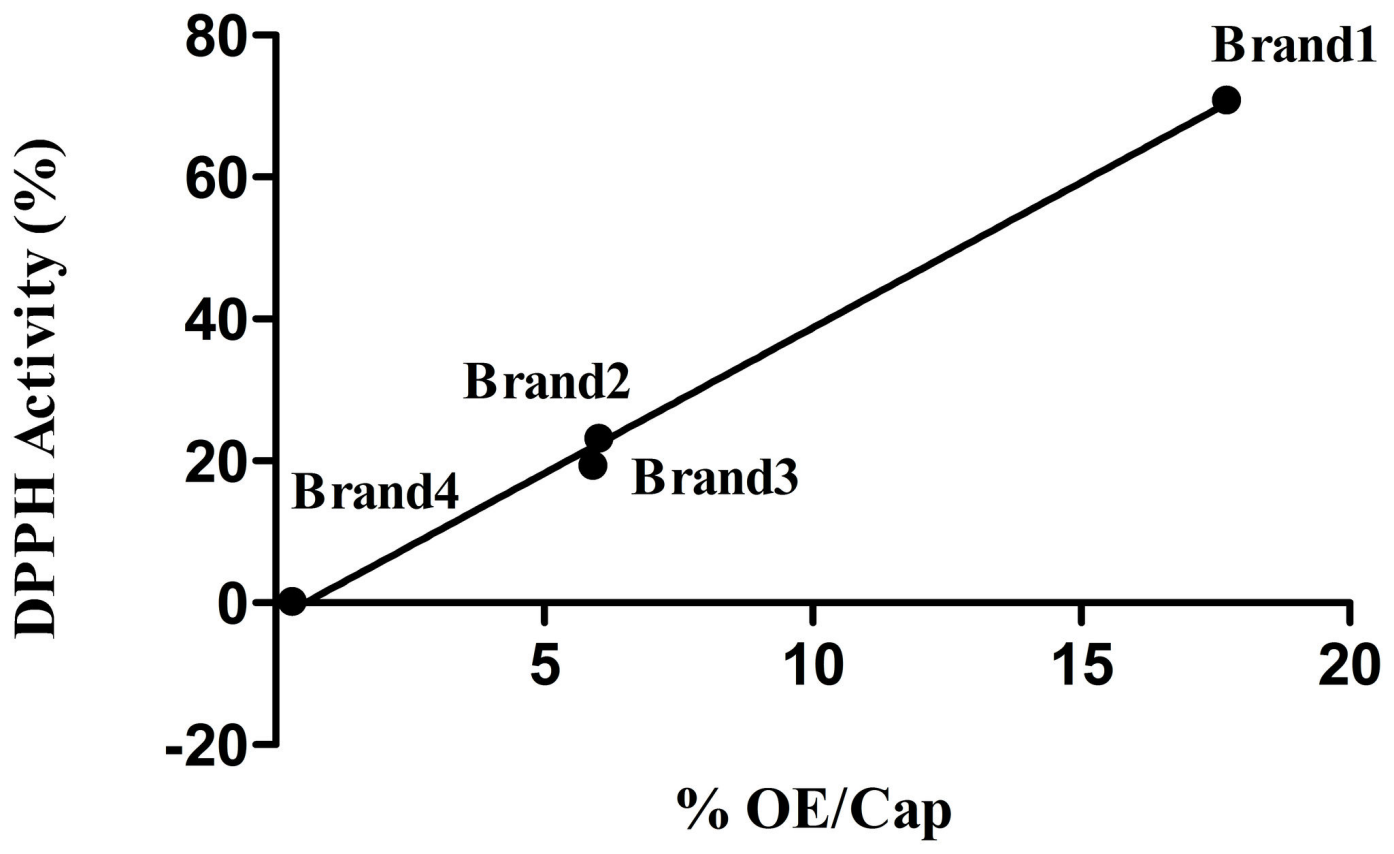
Linear regression of the % OE content in the capsules vs DPPH activity %, (the correlation coefficient was found to be r^2^ = 0.996).

In order to gain insight to the possible antioxidant activity of the different HMP’s, the DPPH radical scavenging method was performed to the list of the examined samples. As it shown in Figure 4 the inhibition caused by B1 samples was found to be higher than the inhibition caused by B2 and B3 samples whereas B4 samples did not cause any inhibition. Specifically, B1 antioxidant capacity was found to be 70.3±1.7, for B2 antioxidant capacity was found to be 16.8±2.6, 21.3±2.1 for B3 and no inhibition was noticed for B4. The results show that the OE content (Figure 4) shows a high linear correlation (DPPH Activity % = 4.1 x % OE/Cap – 2.2, r^2^ = 0.996) to the total antioxidant capacity of the examined samples.

#### MVA Analysis

**Figure 5:**
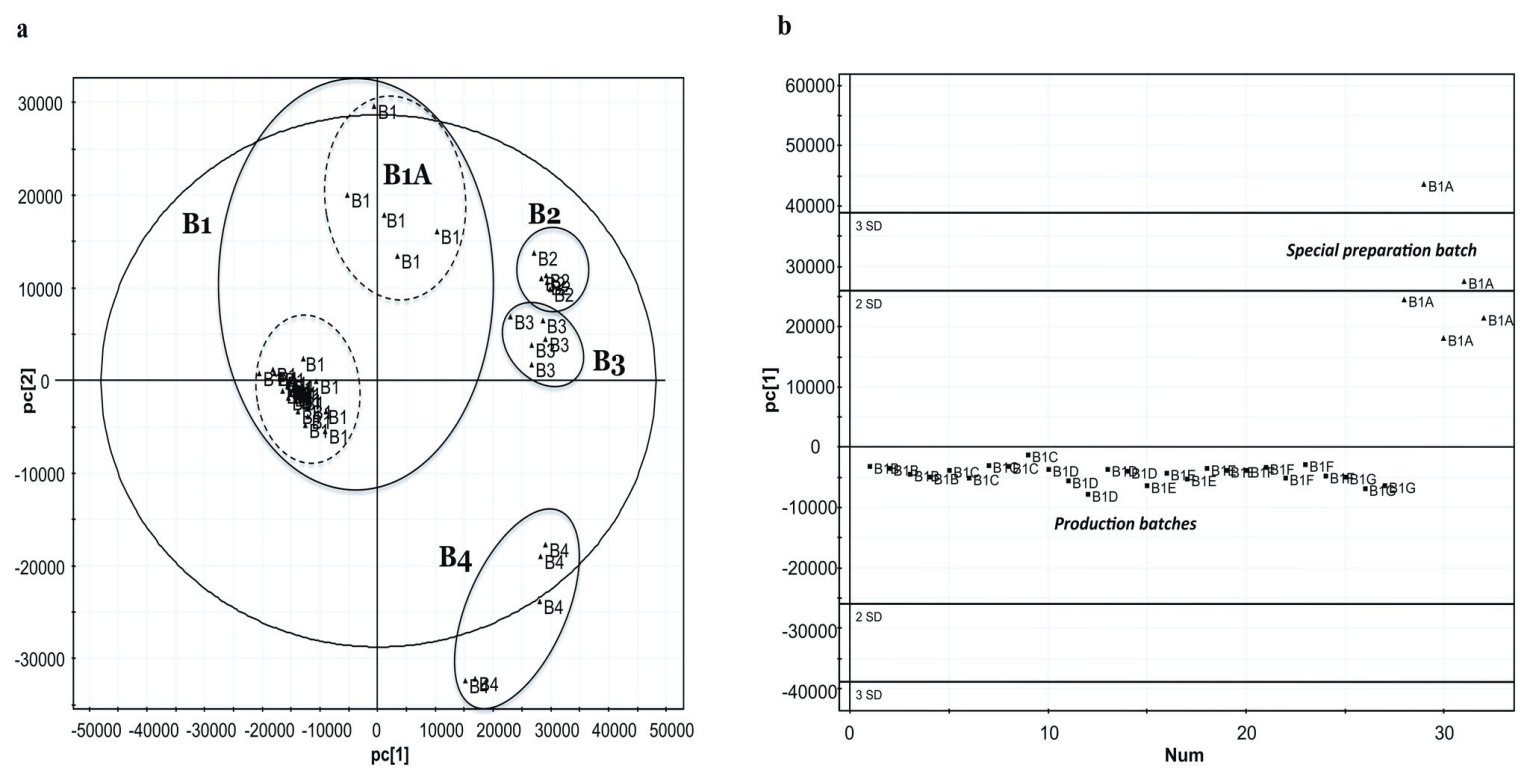
Multivariate analysis of the data obtained from the untargeted quality control approach. **a**. PCA scores plot of the four brands (B1-B4) (n=5 capsules) and the different preparations of B1 (B1A-B1H) (n=5 capsules) obtained after pareto scaling. PCA analysis shows clear grouping of the different examined samples and indicates the excellent batch-to-batch reproducibility obtained from B1, b. PCA based batch to batch reproducibility of the total content of Brand 1. The production batches exhibit low batch to batch variability (<1 SD values) based on their total chemical content. The special preparation batch is separately grouped.

MVA has been employed using the PCA statistical model. Various parameters have been used, such as different scaling e.g. UV or Pareto, and different combinations of Q^2^ and R^2^ in order to assess the number of factors that described as much variance as possible without increasing the complexity of the model excessively. The pareto scaling method has been finally selected as it afforded tighter groups with larger separation. An arbitrary cutoff value of 10% for the explained variance for the pc’s has been employed, in an effort to keep the simplest form for the PCA model. This has been verified by the scree plot, which showed rapid inflection after the second pc. Incorporation of more pc’s led to an insignificant improvement of the model. Thus, only the two first pc’s have been taken into account. In a first approach all samples were included to the dataset, i.e. all samples from all the four Brands i.e five random samples from eight different batches from B1 (B1A – B1H), i.e. seven production batches and one special preparation batch, and in addition five random samples from one batch from the rest of the brands (B2, B3 and B4). The PCA analysis exhibited clear grouping of the different brand samples examined. Thus the scores plot shown in Figure 5a demonstrates the separation of the four brands in different groups applying pareto scaling, which proves the ability of the method to discriminate OE claimed HMP’s from different manufacturers based not only on the target component quantity but also on the overall profile of the extract. The most important metabolites responsible for this grouping (loadings) are summarized in Table S2 as marker ions, corresponding retention times, possible identifications and their peak areas and the relative peak areas. It should be noted that features with identical *m/z* but different retention times possibly correspond to isomers. Furthermore, an analysis for possible adducts (M-H2O-H, M-H, M+Na-2H, M+Cl, M+K-2H, M+FA-H, M+Br, 2M-H, 2M+FA-H) has been performed for all the features in order to verify the possibility of occurrence of chromatographic peaks due to adduct formation. The first pc holds for the largest part of variation (30.8%) between the different brands, while the second pc holds for the 11.1% of the variation.

As described before the second major target of the current work was to examine the quality of the production procedure of HMP’s, based on the uniformity of the contained minor substances profile and not only taking into account the content of the active label substance OE for establishing the batch-to-batch reproducibility of the finished products. Such an approach will ensure the proper, within specifications, pharmacological activity of the HMP’s respecting possible synergism concerns. This can be assured not only residing in the content uniformity concerning the concentration of major compound, but also controlling the amounts of the minor constituents between bathes. Hence, concerning the Brand B1, eight different batches were sampled and analyzed by the described UHPLC-HRMS methodology in order to assess the batch-to-batch reproducibility based on their total chemical content. Figure 5b shows only the B1 samples (production batches and the special preparation batch) for reasons of clarity, i.e. the MVA is not confounded by the presence of the other brands, which could potentially lead to tighter grouping of the production batches due to the large separation distance from the other batches in pc1 as can be seen in Figure 5a. It is clear that all data points from the production batches lie within one SD from the mean, whereas the special preparation batch lies near or above the 2 SD limit. This reflects the excellent batch-to-batch reproducibility obtained from the production batches of B1 and furthermore it proves that the even a slightly different preparation (B1A) from the same production line (B1B-B1H) could be distinguished. Thus it seems that any “out-of-specification” batches could be easily detected. It is also evident from Figure 5a that the different preparations of B1 (B1A vs. B1B-B1H) can separate according to the second principal component (pc2) whereas in the first principal component (pc1) they seem indistinguishable, proving that slight differences play a critical role in the grouping of them. Overall it could be reasonably proposed that the quality of HMP’s could be divided in two parts the first one concerning the active ingredient content whereas the second part should involve the reproducibility of the overall production reflecting the total quality of the extract used as well as the production procedures. Thus extracts from different sources or undergone different production procedures (e.g. longer storage, different storage temperature, differentiated production procedures etc.) could potentially lead to essentially different products from the pharmacological point of view. In the current work a workflow is proposed in order to overcome such issues. 

## Conclusion

Overall an LC-(-ESI)-MS method in the SIM mode for the quantification of OE and its main degradation product, HT, in different selected commercial OE claimed HMP’s was developed and validated according to the ICH requirements. Despite the fact that SRM mode in MS is considered as more sensitive and selective, such instrumentation is rather expensive and complicated. Thus, an SRM based methodology is not fit of purpose for the current analysis, considering also the fact that the targeted compounds are present in large amounts to the HMP’s under investigation. Results regarding specificity, recovery, matrix effect, regression model, quantitation and detection limit, repeatability, intermediate precision, accuracy and robustness are satisfactory, and the developed method is applicable for its intended purpose. Furthermore, a simple and inexpensive sample preparation procedure followed by a fast analytical run of 3.1 min (UHPLC-like analysis on a Fused Core column) to analyze and quantify all analytes as well as the IS has been employed. Unlike the common practice, the matrix of the OE claimed HMP has been isolated and used in order to assess critical parameters such as the matrix effect; a crucial parameter that should be always considered when dealing with MS based detection.

The measured content of OE in the preparation B1 was in agreement with the concentration reported on label, and confirmed the validity of the developed method. In contrast, preparation B2 and B3 were out of specification whereas in B4 OE was found to be completely absent. In fact three out of four preparations were not compliant to the specifications, thus verifying the need of new validated methodologies for the quality control of HMP’s.

Additionally a UHPLC-(-ESI)-HRMS-based metabolomics-like approach was developed in order to evaluate the holistic quality of the four different brands of commercial OE claimed HMP’s. By this approach, certain quality issues of commercial OE claimed HMP’s were revealed. The data obtained by the present investigation led to a satisfactory and quick characterization methodology of complex mixtures of HMP’s. Nevertheless, it should be noted that UHPLC-(-ESI)-HRMS-based profiling is more sensitive but less reproducible than its NMR counterpart, which is less sensitive but more reproducible. Thus, NMR based profiling could be an attractive alternative in this issue.

This work could potentially consist the basis for further investigation on the quality control and the stability of commercial HMP’s. Concluding the proposed methodology can be used for routine quantitative analysis, quality control as well as for studies on the production process of HMP’s. Overall, the proposed methodology should be incorporated to the production process of HMP’s or even pharmaceuticals in a long-term manner. Such an approach will enhance the confidence and will reassure the high quality of the final products. 

## Supporting Information

Table S1 **Targeted analysis data.** Peak areas of OE, HT and IS in the examined HMP’s and quantification thereof by the proposed LC-SIM methodology.
(XLS)

Table S2 **The most important loadings (metabolites) i.e. marker ions, corresponding retention times, possible identifications (as determined from an in-house substance library) and their peak areas and relative peak areas (%), responsible for the grouping of the four different brands of HMP’s.**
(XLSX)

## References

[1] Agalias A, Melliou E, Magiatis P, Tsarbopoulos A, Mitaku S (2005) Quantitation of polyphenols and secoiridoids in decoctions of *Olea* *europaea* leaves from ten Greek cultivated varieties. J Liq Chromatogr 28: 1557–1571. doi:10.1081/JLC-200058355.

[2] Romani N, Mulinacci P, Pinelli F, Vincieri FF, Cimato A (1999) Polyphenolic content in five tuscany cultivars of *Olea* *europaea* L. J Agric Food Chem 47: 964-967. doi:10.1021/jf980264t. PubMed: 10552399. 10552399

[3] Japon-Lujan R, Luque de Castro MD (2006) Superheated liquid extraction of oleuropein and related biophenols from olive leaves. J Chromatogr A 1136: 185–191. 17045596 10.1016/j.chroma.2006.09.081

[4] Renaud S, De Lorgeril M, Delaye J, Guidollet J, Jacquard F et al. (1995) Cretan Mediterranean diet for prevention of coronary heart disease. Am J Clin Nutr 6: 1360–1367. PubMed: 7754988. 10.1093/ajcn/61.6.1360S 7754988

[5] Galli C, Visioli F (1999) Antioxidant and other activities of phenolics in olives/olive oil, typical components of the mediterranean diet. Lipids 34: 23-26. doi:10.1007/BF02562224. PubMed: 10188593. 10419084

[6] Kontogianni VG, Gerothanassis IP (2012) Phenolic compounds and antioxidant activity of olive leaf extracts. Nat Prod Res 26: 186–189. doi:10.1080/14786419.2011.582842. PubMed: 22060136. 22060136

[7] Pereira AP, Ferreira ICFR, Marcelino F, Valentao P, Andrade PB et al. (2007) Phenolic compounds and antimicrobial activity of olive (*Olea* *europaea* L. Cv. Cobrancosa) leaves. Molecules 12 (5): 1153–1162. 17873849 10.3390/12051153 PMC6149345

[8] Omar SH (2010) Cardioprotective and neuroprotective roles of oleuropein in olive. 18: 111–121. doi:10.1016/j.jsps.2010.05.005. PubMed: 23964170. PMC3730992 23964170

[9] Keys A (1997) Coronary heart disease in seven countries.1970. Nutrition 13(3): 249-253. doi:10.1016/S0899-9007(97)91292-2. PubMed: 9131697. 9131696

[10] Andreadou I, Iliodromitis EK, Mikros E, Constantinou M, Agalias A et al. (2006) The olive constituent oleuropein exhibits anti-ischemic, antioxidative, and hypolipidemic effects in anesthetized rabbits. J Nutr 136: 2213–2219. PubMed: 16857843. 16857843 10.1093/jn/136.8.2213

[11] Visioli F, Galli C (2001) Antiatherogenic components of olive oil. Curr Atheroscler Rep 3: 64–67. doi:10.1007/s11883-001-0012-0. PubMed: 11123850. 11123850

[12] Scoditti E, Calabriso N, Massaro M, Pellegrino M, Storelli C et al. (2012) Mediterranean diet polyphenols reduce inflammatory angiogenesis through MMP-9 and COX-2 inhibition in human vascular endothelial cells: A potentially protective mechanism in atherosclerotic vascular disease and cancer. Arch Biochem Biophys 527: 81-89. doi:10.1016/j.abb.2012.05.003. PubMed: 22595400. 22595400

[13] Poudyal H, Campbell F, Brown L (2010) Olive Leaf Extract Attenuates Cardiac, Hepatic, and Metabolic Changes in High Carbohydrate High Fat Fed Rats. J Nutr 140: 946–953. doi:10.3945/jn.109.117812. PubMed: 20335636. 20335636

[14] Jemai H, El Feki A, Sayadi S (2009) Antidiabetic and Antioxidant Effects of Hydroxytyrosol and Oleuropein from Olive Leaves in Alloxan Diabetic Rats. J Agric Food Chem 57(19): 8798-8804. doi:10.1021/jf901280r. PubMed: 19725535. 19725535

[15] Acquaviva R, Di Giacomo C, Sorrenti V, Galvano F, Santangelo R et al. (2012) Antiproliferative Effect Of Oleuropein In Prostate Cell Lines. Int J Oncol 41(1): 31-38. PubMed: 22484302. 22484302 10.3892/ijo.2012.1428

[16] European Medicines Agency (2011) Assessment Report on Olea europaea L, folium. Based on Article 16d(1), Article 16f and Article 16h of Directive 2001/83/EC as amended (traditional use). Assessment report on Olea europaea L., folium, EMA/HMPC/430506/2009. EMA website. Available: http://www.ema.europa.eu/docs/en_GB/document_library/Herbal_-_HMPC_assessment_report/2012/04/WC500125459.pdf . Accessed 2013 Oct 3

[17] Granados-Principal S, Quiles JL, Ramirez-Tortosa CL, Sanchez-Rovira P, Ramirez-Tortosa MC (2010) Hydroxytyrosol: from laboratory investigations to future clinical trials. Nutr Rev 68(4): 191-206. doi:10.1111/j.1753-4887.2010.00278.x. PubMed: 20416016. 20416016

[18] (2004) Directive 2004/24/EC of the European Parliament and of the Council, amending, as regards traditional herbal medicinal products, Directive 2001/83/EC on the Community code relating to medicinal products for human use. Official Journal of the European Union L 136-85. Available: http://eur-lex.europa.eu/LexUriServ/LexUriServ.do?uri=OJ:L:2004:136:0085:0090:en:PDF.

[19] http://www.ema.europa.eu/ema/index.jsp?curl=pages/regulation/general/general_content_000208.jsp&mid=wc0b01ac05800240cf.

[20] Williamson EM et al. (2001) Synergy and other interactions in phytomedicines.Phytomedicine 5: 401-409. PubMed: 11695885. 10.1078/0944-7113-00060 11695885

[21] Dierkes G, Krieger S, Dück R, Bongartz A, Schmitz OJ et al. (2012) High-Performance Liquid Chromatography−Mass Spectrometry Profiling of Phenolic Compounds for Evaluation of Olive Oil Bitterness and Pungency. J Agric Food Chem 60: 7597−7606. doi:10.1021/jf3020574. PubMed: 22804690. 22804690

[22] Zoidou E, Melliou E, Gikas E, Tsarbopoulos A, Magiatis P et al. (2010) Identification of Throuba Thassos, a Traditional Greek Table Olive Variety, as a Nutritional Rich Source of Oleuropein. J Agric Food Chem 58: 46–50. 19957933 10.1021/jf903405e

[23] Sanchez JL, Giambanelli E, Quirantes-Piné R, Cerretani L et al. (2011) Wastes Generated during the Storage of Extra Virgin Olive Oil as a Natural Source of Phenolic Compounds. J Agric Food Chem 59: 11491–11500. doi:10.1021/jf202596q. PubMed: 21939275. 21939275

[24] Bazoti FN, Gikas E, Tsarbopoulos A (2010) Simultaneous quantification of oleuropein and its metabolites in rat plasma by liquid chromatography electrospray ionization tandem mass spectrometry 24. Biomed Chromatogr pp. 506–515. 10.1002/bmc.1319 19795379

[25] Ortega-García F, Peragón J (2010) HPLC analysis of oleuropein, hydroxytyrosol, and tyrosol in stems and roots of *Olea* *europaea* L. cv. Picual during ripening. J Sci Food Agric 90: 2295–2300. doi:10.1002/jsfa.4085. PubMed: 20648529. 20648529

[26] Tan HW, Tuck KL, Stupans I, Hayball PJ (2003) Simultaneous determination of oleuropein and hydroxytyrosol in rat plasma using liquid chromatography with fluorescence detection. 785: 187–191 doi:10.1016/S1570-0232(02)00855-3. PubMed: 12535851. 12535851

[27] Zhou T, Qian T, Wang X, Li X, Cao L et al. (2011) Application of LC-MS/MS method for the in vivo metabolite determination of oleuropein after intravenous administration to rat. Biomed - Journal of Chromatogr 25(12): 1360-1363. doi:10.1002/bmc.1609. PubMed: 21308708. 21308708

[28] Briante R, Patumi M, Terenziani S, Bismuto E, Febbraio F et al. (2002) *Olea* *Europaea* L. Leaf Extract And Derivatives: Antioxidant Properties. J Agric Food Chem 50: 4934−4940. doi:10.1021/jf025540p. PubMed: 12166985. 12166985

[29] Aturki Z, Fanali S, D’Orazio G, Rocco A, Rosati C (2008) Analysis of phenolic compounds in extra virgin olive oil by using reversed-phase capillary electrochromatography. Electrophoresis 29: 1643–1650. doi:10.1002/elps.200700547. PubMed: 18383030. 18383030

[30] Aouidi F, Dupuy N, Artaud J, Roussos S, Msallem M et al. (2012) Rapid quantitative determination of oleuropein in olive leaves (*Olea* *europaea*) using mid-infrared spectroscopy combined with chemometric analyses. Industrial Crops and Products 37: 292–297. doi:10.1016/j.indcrop.2011.12.024.

[31] International Conference On Harmonisation Of Technical Requirements For Registration Of Pharmaceuticals For Human Use (2010) ICH Harmonised Tripartite Guideline Validation Of Analytical Procedures: Text And Methodology Q2 (R1). Current Step 4 version Parent Guideline dated 27 October 1994 (Complementary Guideline on Methodology dated 6 November 1996 incorporated in November 2005). ICH website. Available: http://www.ich.org/fileadmin/Public_Web_Site/ICH_Products/Guidelines/Quality/Q2_R1/Step4/Q2_R1__Guideline.pdf . Accessed 2013 Oct 3.

[32] Yang P, Litwinski GR, Pursch M, McCabe T, Kuppannan K (2009) Separation of natural product using columns packed with Fused-Core particles. J Sep Sci 32: 1816–1822. 19425022 10.1002/jssc.200900005

[33] Manchón N, D’Arrigo M, García-Lafuente A, Guillamón E, Villares A et al. (2010) Fast analysis of isoflavones by high-performance liquid chromatography using a column packed with fused-core particles. Talanta 82(5): 1986–1994. doi:10.1016/j.talanta.2010.08.050. PubMed: 20875606. 20875606

[34] Orozco-Solano MI, Ferreiro-Vera C, Priego-Capote F, de Castro Luque (2012) Automated method for determination of olive oil phenols and metabolites in human plasma and application in intervention studies. J Chromatogr A 1258: 108–116. doi:10.1016/j.chroma.2012.08.057. PubMed: 22944382. 22944382

[35] Hadad GM, Abdel Salam RA, Emara S (2012) Validated and optimized high-performance liquid chromatographic determination of tizoxanide, the main active metabolite of nitazoxanide in human urine, plasma and breast milk. J Chromatogr Sci 50(6): 509-515. doi:10.1093/chromsci/bms041. PubMed: 22525879. 22525879

[36] Lan K, Zhang Y, Yang J, Xu L (2010) Simple quality assessment approach for herbal extracts using high performance liquid chromatography-UV based metabolomics platform. J Chromatogr A 1217: 1414–1418. doi:10.1016/j.chroma.2009.12.031. PubMed: 20045112. 20045112

[37] Zhang HM, Li SL, Zhang H, Wang Y, Zhao ZL et al. (2012) Holistic quality evaluation of commercial white and red ginseng using a UPLC-QTOF-MS/MS-based metabolomics approach. J Pharm Biomed Anal 62: 258–273. doi:10.1016/j.jpba.2012.01.010. PubMed: 22310552. 22310552

[38] Agalias A, Magiatis P, Skaltsounis AL, Mikros E, Tsarbopoulos A et al. (2007) A New Process For The Management Of Olive Oil Mill Waste Water And Recovery Of Natural Antioxidants. J Agric Food Chem 55: 2671−2676. doi:10.1021/jf063091d. PubMed: 17348673. 17348673

[39] Stagos D, Portesis N, Spanou C, Mossialos D, Aligiannis N et al. (2012) Correlation of total polyphenolic content with antioxidant and antibacterial activity of 24 extracts from Greek domestic Lamiaceae species. Food Chem Toxicol 50(11): 4115–4124. doi:10.1016/j.fct.2012.08.033. PubMed: 22939934. 22939934

